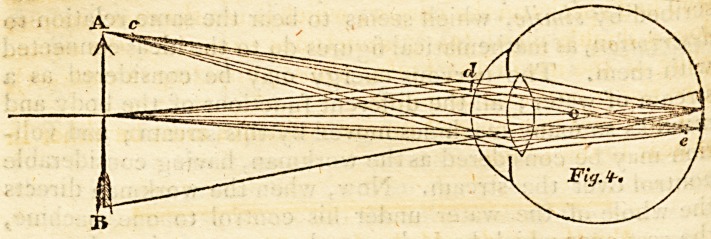# On Distinct Vision at Different Distances

**Published:** 1818-09

**Authors:** Nicholas Littleton


THE LONDON

### Medical and Physical Journal.

3 OF VOL. XL.] SEPTEMBER, 1818. [NO. 235.

"For many fortunate discoveries in medicine, and for the detection of nume-
"rous errors, the world is indebted to the rapid circulation of Monthly
"Journals; and there never existed any work to which the Faculty in
"EUROPE and AMERICA were under deeper obligations than to the
"Medical and Physical Journal of London, now forming a long, but an
"invaluable, series."—RUSH.

*For the London Medical and Physical Journal.*

### *On distinct Vision at different Distances*; by NICHOLAS LITTLETON, Esq.

MANY have accounted for the means by which we are enabled to see distinctly, at such different distances, as half a foot to three feet, but I rather think no one as yet is fully satisfied with regard to it.

It has been conjectured that there is a muscular power in the fibres of the crystalline lens itself, by which it may be enabled to assume a more or less globular form, accordingly as the object viewed is at a greater or less distance. Some have thought that it was effected by a muscular power in the ciliary processes, by which it was possible for the lens to be drawn a little farther from the retina, whilst viewing near objects. Others have supposed it was effected through the agency of the muscles, exterior to the globe of the eye. And, lastly, a few have thought it possible for the iris to effect it. These four different means are all that have been thought of which are applicable to the human eye, wherein there is no marsupium, nor any muscular power, by which the cornea can be made to approach nearer to the lens.

For me to mention the authors of these different conjectures, or to state objections as to the probability, or even possibility, of their truth, would be to extend my paper to the length of an essay more fitted for separate publication, than for admission in your Journal, which I consider as intended for original matter, and not for the discussion of hacknied topics, with which all thinking minds must be already acquainted. Students, however, may refer to Dr. Porterfield's Essays in the Medical Facts and Observations; which, although containing some error, are ingeniously written; so ingeniously, that I hope some remarks on these essays may hereafter be admitted in your Journal.

When I wrote on the iris, it will appear I was of opinion that, for distinct vision, it was not necessary that there should be a perfect image formed on the retina; such opinion arose from an inability of possibly accounting for such perfect images, at different distances; notwithstanding, we all know that we can see distinctly at such distances; therefore, it was thought vision might be distinct, although the image was somewhat imperfect. But that we should see so distinctly when an imperfect image is formed on the retina, as when a perfect image is formed, was unreasonable, and it afforded satisfaction to be able to remove this irrationality.

It is unnecessary for me to give an anatomical account of the eye, as enough on that head may be read in any system of anatomy. All who read this know we have a retina, and it must be equally plain that this retina must have some thickness; this thickness is not easily ascertained in so soft a substance, and so difficult to be got at, without in some measure disturbing its connexion with the contiguous parts. I should think that its thickness may be about one-fiftieth part of an inch. The retina when seen in the dead body is slightly opake; but all membranes lose their transparency by death, and the more so, as their consistence is less firm. What then must be the loss of transparency in so soft a substance as the retina? Therefore the retina will be considered as a transparent substance one-fiftieth part of an inch thick.

There is no reason to deny that the retina is equally capable of receiving the impression of the rays of light through the whole of its thickness. Physiologists heretofore have only considered it as receiving impressions on its anterior or central surface; but, as its substance is transparent, therefore, although the rays should not have come to a focus exactly at this surface, yet the rays can pass on, and come to a focus behind this surface, somewhere in the substance of the retina, which is considered as extending one-fiftieth of an inch backwards. To illustrate this, fig. 1. is subjoined. The figure is smaller than the human eye;* but that is of no consequence with regard to the principle. The whole globe is made eight-tenths of an inch in diameter. The anterior curve of the crystalline lens has a radius of 2·5 tenths of an inch. The rays proceeding from an object at A, two inches off, are refracted by the lens to a focus at *a*, in the substance of the retina; but rays proceeding from B, 6 inches off, and also the parallel rays from C, come to a focus at *c* and *b*, be-

* We have been obliged to reduce the drawings of Mr. Littleton's figures proportionally to the width of our pages.—EDITORS.

fore they arrive at the retina, and therefore are not seen distinctly.

For facility in calculation, no account is taken of the refraction at the cornea, or at the vitreous humour; but the whole refraction of the humours together is placed in the lens. The refraction from air into water, as at the cornea, is found to be about 4 to 3 of water into glass, as at the anterior surface of the lens may be as 13 to 12, and of glass into water, as at the vitreous humour, $\frac{13}{12}$, making together $\frac{2}{3}$, which has been fixed on for calculation, as being near the truth. Now, suppose the object viewed is at a distance of 6 inches from the lens, how far behind the refracting surface will the rays proceeding from such object come to a focus?

The radius of the lens is fixed at 2.5 tenths of an inch, and it is assumed that the sine of the angle of refraction is two-thirds of the sine of the angle of incidence; or O G,* fig. 2, is equal to two-thirds of D E. Suppose also that the bases of the pencils of rays extend over an area of globular surface, equal to 96°, which is as much as is the extent of the anterior curved surface of the lens. By referring to fig. 2, we have C O = 2·5, A O = 62·5, and the angle at C O A, half the angle C O F; the whole lenticular curve is therefore equal to 48°. Hence we have the case in trigonometry, where the two sides A O and O C are given in tenths of an inch, and their included angle

| | |
|---|---|
| 1·7781513 | is the logarithm of 60, the difference of the sides, |
| 10·3514169 | logarithmic tangent of 66°, half the unknown angles. |
| 8·1870866 | arithmetical complement of the log. of 65, the sum of the sides. |
| 10·3166548 | log. tangent of 64° 15′, half the difference of the angles. |

66° — 64° 15′ = 1° 45′, the angle C A O, and 48° + 1° 45′ = 49° 45′, the angle O C H, two-thirds of the natural sine, of which is·5988216 the sine of 30° 25′, which is the angle O C B,

| | |
|---|---|
| 9·7065394 | logarith. sine of 30° 25′, the angle O C B. |
| ·3979400 | logarith. of 2·5, the side C O. |
| ·5238666 | arith. comp. of the log. sine of 17° 25′, the angle C B O. |
| ·6283460 | |

Logarithm of 4·249, the distance from O, the centre of the lenticular curve, to B, the focus or point where the rays meet. Which distance O B, added to the radius O C, gives the answer to the proposed question. After the same manner, the distance O B may be found, where the distance of the object from the lens is greater or less than 6 inches.

Several results of such calculations it may not be amiss to add.

When the object is only two inches distant from the lens, the distance from O to B will be found

| | | | |
|---|---|---|---|
| | 4·9 tenths. | At 24 inches | 4·022 |
| At $2\frac{1}{4}$ it is | 4·785 | 36 | 3·994 |
| $2\frac{1}{2}$ | 4·7 | And for parallel rays | 3·944 tenths. |
| 3 | 4·314 | | |
| 6 | 4·249 | | |

Thus, when rays proceeding from an object 2 feet off come to a focus on the anterior or central surface of the retina, then also rays proceeding from an object only 6 inches distant may come to a focus in the substance of the retina, as the difference between 4·249 and 4·022 tenths of an inch is only about one-fiftieth of an inch. But in myopes the range of vision is only half an inch instead of 18 inches, as the difference between 4·9 and 4·7 tenths is also one-fiftieth of an inch. And presbi, who see at the nearest distance of two or three feet, would see at all distances beyond this distinctly, were they not prevented from seeing perfectly; 1st, From the size of the angle C A F, fig. 2, (the angle of vision) being less than half a minute or a minute. 2ndly, From the weakness of the impression arising from the divergency of the rays of light, whereby but few rays can impinge on the retina. And, 3rdly, From some of the rays being intercepted by the want of perfect transparency in the atmosphere.

For calculation, the two rays A O and A C have alone been attended to. But, as there is an indefinite number of rays in each pencil, which for the most perfect vision must all be brought to one focus, therefore there is a greater consistence of the lens towards its centre, by which the refraction is proportionally increased as the rays fall nearer to the centre. On the contrary, if the lens had been uniformly consistent, the rays would not all meet in one point, but would have been scattered to some length on the line O B, fig. 3; as those rays falling on the edge of the lens would come to a focus before the rays which fall nearer to the centre.

Hence it is shewn why it is that we can see objects at different distances at the same time; although some physiologists would persuade themselves that they cannot, still every body knows it is the case, when their attention is equally directed to such objects. It is true that the attention may be so taken up with viewing a single object, that all others, whether at the same or different distances, may pass unnoticed; but all the other senses may also, during this extreme attention, remain inactive, and even hunger and bodily pain may pass unnoticed likewise. Blindness, therefore, to all but what is attended to, does not arise from any muscular conformation in or about the globe of the eye, but from a principle of the mind, which can better be described by *simile,* which seems to bear the same relation to *description,* as mathematical figures do to the ideas connected with them. The nervous energy may be considered as a stream of water; all the different functions of the body and mind as so many machines moved by this stream; and volition may be considered as the workman, having considerable control over the stream. Now, when the workman directs the whole of the water under his control to one machine, the rest over which he had control must stop: just the same is the blindness to all things to which we do not attend.

This persuasion to opinion formed on false reasoning, though even contrary to glaring facts, is not uncommon in philosophy, but more especially belongs to such systems, where reason is not allowed its full weight. A blame of the sort may not be misapplied to the writer, but he is assured that it does not arise from repugnance to reasoning on any subject, but rather from inadvertency or ignorance. Therefore, hoping that this letter may be approved of,

He remains, &c.

*Padstow; July.* NICHOLAS LITTLETON.

The two rays A C, A D, of a cone or pencil of rays, proceeding from the point A, fall on the curved surface of the lens E F. The ray A C, falling on the edge of the lens, is rendered convergent, and has its focus at B; but the ray A D, falling near the centre, is only rendered parallel, and after refraction proceeds in the direction D G.

In No. 231, I made some remarks on a letter of Dr. Adams's, written on a subject that had been discussed by the London Medical Society, but it seems not fully to his satisfaction. In the former letter all the reasonings are conducted on the principle adopted by Dr. A. not because such principles are true, but because it was thought proper, inasmuch as there is a mere possibility that the principle may be correct. Now the endeavour will be to shew that the principle itself is false. It is assumed that an opake body on the lens can cause the appearance of a dark spot. How astonishing! that a whole society should inadvertently overlook the almost impossibility of such a cause of *muscæ volitantes*, which oversight no doubt occurred, otherwise Dr. A. would never have published his letter. If a light be placed behind a lens, it will be found that the interposition of an opake body, any way near to the refracting surfaces, will not cause a dark spot on the refracted image, but only cause the whole image to be more obscure. In order to cause a dark spot on the image, the opake body must be interposed, either near to the body whose image is to be refracted, or near to the refracted image, so as to obstruct the passage of the rays, forming a whole pencil or cone; as in fig. 4, a small body at *c* or *e* obstructs the passage of a whole pencil, which small body at *d* would only obstruct some of the rays, and that of every pencil proceeding from the whole length of the object A B; hence the whole image would only be rendered more obscure. Just such obscurity of vision exists in people who have specks on their cornea, or who have incipient cataract: those say objects appear as through smoke, or through a mist.

The bare possibility to which I have alluded, that the principle of Dr. A. may be correct, rests on the following weak grounds. Is it not possible that that part of the object

I

which has its rays falling nearly in a continuous line with the radius of the refracting curve, as do those at and near to A O? Is it not possible that the obstruction of these rays in particular may cause an appearance of a dark spot? I think not, as, then, every one would have an appearance of muscæ who had specks on the cornea, which is not the case; neither does a double pupil (as is sometimes caused by the entangling of the iris in the knife, whilst extracting a cataract), give the appearance of muscæ.

The cause therefore of muscæ volitantes must be either in or near to the retina.

---

*For the London Medical and Physical Journal.*

*Nosological Strictures;* by T. PARKINSON, M.D.

## Figures and Tables

**Fig. 1. f1:**
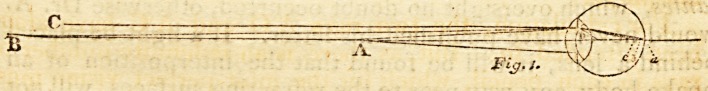


**Fig. 2. f2:**
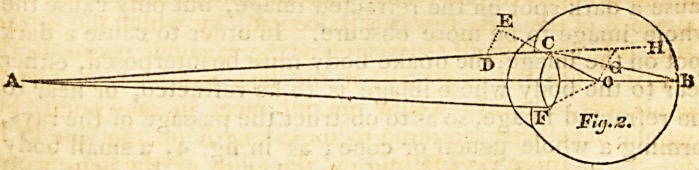


**Fig. 3. f3:**
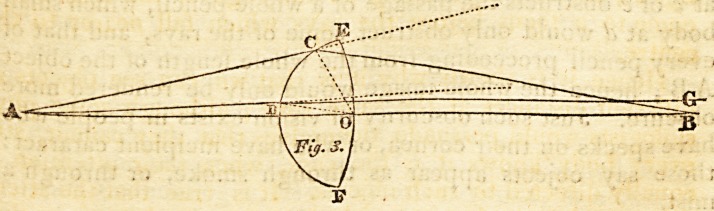


**Fig. 4. f4:**